# MiR-486 as an effective biomarker in cancer diagnosis and prognosis: a systematic review and meta-analysis

**DOI:** 10.18632/oncotarget.24189

**Published:** 2018-01-12

**Authors:** Min Jiang, Xuelian Li, Xiaowei Quan, Xianglin Yang, Chang Zheng, Xia Hao, Ruoyi Qu, Baosen Zhou

**Affiliations:** ^1^ Department of Epidemiology, School of Public Health, China Medical University, Shenyang, China; ^2^ Key Laboratory of Cancer Etiology and Prevention (China Medical University), Liaoning Province Department of Education, Shenyang, China; ^3^ Department of Clinical Epidemiology, First Affiliated Hospital, China Medical University, Shenyang, China

**Keywords:** miR-486, cancer, meta-analysis, diagnostic, prognosis

## Abstract

**Purpose:**

MiR-486 was found to be associated with cancer’s diagnosis and prognosis. This meta-analysis aimed to investigate the potential effect of miR-486 on cancer detection and prognosis.

**Materials and Methods:**

We searched PubMed, Cochrane library, Embase, Chinese National Knowledge Infrastructure (CNKI) and Wanfang databases to find all correlated articles. The STATA 11.0 was applied to estimate the pooled effects, heterogeneity and publication bias.

**Results:**

The pooled sensitivity (SEN), specificity (SPE) and Area under the curve (AUC) were 82% (95% CI: 78–85%), 88% (95% CI: 83–92%) and 0.91 (95% CI: 0.88–0.93). Subgroup analysis indicated miR-486 from circulating samples exhibited higher diagnostic accuracy with the AUC was 0.90 (95% CI: 0.87–0.92) than miR-486 from other specimen with the AUC of 0.78 (95% CI: 0.75–0.82) and miR-486 obtained a better diagnostic value in the Asian population with the AUC of 0.94 (95% CI: 0.91–0.95) than the Caucasian and Caucasian/African population with the AUC of 0.80 (95% CI: 0.76–0.83) and 0.89 (95% CI: 0.86–0.91) respectively. MiR-486 obtained high value for the diagnosis of non-small cell lung cancer with SEN, SPE and AUC were 0.82 (95% CI: 0.0.77–0.87), 0.90 (95% CI: 0.84–0.94) as well as 0.92 (95% CI: 0.89–0.94) respectively. For the 7 prognostic tests, the pooled hazard ratio (HR) was 0.48 (95% CI: –0.13–1.08) for low versus high miR-486 expression.

**Conclusions:**

This meta-analysis indicated that miR-486 can be used as ideal biomarkers in the cancer’s diagnosis. However, Low miR-486 expression did not increase the risk of poor outcome.

## INTRODUCTION

MicroRNA is a group of 19–22 nucleotide, small, single-stranded and conserved non-coding RNA that acts as a regulator of gene expression at both the post-transcriptional and the translational levels through acting on the 3’-untranslated region (UTR) of messenger RNA (mRNA) [1]. MicroRNAs involve in various biological processes associated with the tumorigenesis such as the cellular proliferation, differentiation, metabolism as well as apoptosis [2, 3]. It is available to isolate the microRNAs from the clinical specimens including the plasma, serum, sputum and tissue. Meanwhile, it has a high stability. Due to these advantages, the microRNAs are increasingly becoming an ideal tool for the detection of human cancer.

Aberrant expression of miR-486 (miR-486-5p) has been reported to be associated with different types of human cancer such as hepatocellular carcinoma (HCC) [4, 5], lung cancer [6], breast cancer [7], esophageal squamous cell carcinoma (ESCC) [8] and pancreatic cancer (PC) [9, 10]. It can act as both the tumor suppressor and oncogene to participant in the development and progression of tumors. The down-regulation of miR-486 can promote the progression of lung cancer [6], HCC [4, 5], breast cancer [7] and osteosarcoma [11], while it is usually up-regulated in PC [9, 10], chronic myeloid leukemia [12] and gliomas [13]. Recently, a series of articles have identified that miR-486 might be applied as a biomarker for cancer detection and prognosis. However, as a result of the small sample sizes, different microRNA profiling and the differences of the specimen and ethnicity, many articles showed conflicting conclusions and no meta-analysis was conducted to explore the association between miR-486 and diagnosis as well as the prognosis of human cancer. Therefore, this meta-analysis was performed to assess the performance of miR-486 in the detection and prognosis for human cancer.

## RESULTS

### Literature search and the studies’ characteristics

**Figure 1:**
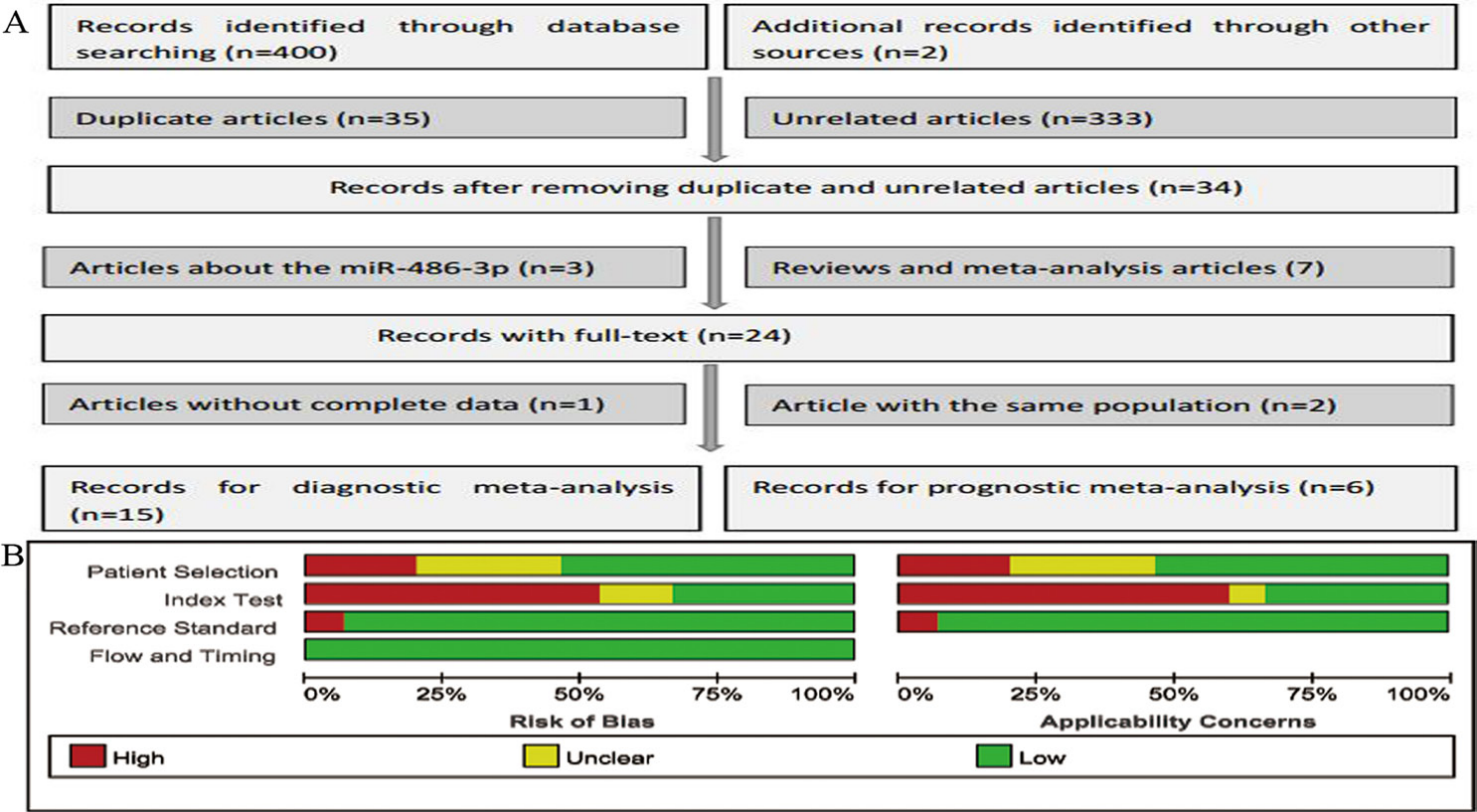
Flow chart of this meta-analysis of miR-486 in cancer detection (**A**) and the quality of these included articles according to the QUADAS-2 guidelines: proportion of articles with risk of bias (left) and proportion of articles with regarding applicability (right) (**B**).

As described in Figure 1A, based on the primary literature research, 402 eligible articles were included, of which 368 articles were removed as duplicate and unrelated articles. And then 7 reviews and 3 articles about miR-486-3p were also excluded, leaving 24 articles with full texts. After carefully reading, another 3 articles were then removed: 1 article without complete data and 2 articles with the same population with other articles. Ultimately, 21 articles [14–33] with 29 studies were published from 2010 to 2017. 15 articles [14–28] with 22 studies were about the value of miR-486 for cancer detection while the remaining 6 articles [29–33] with 7 studies were about the cancer prognosis.

### Diagnostic meta-analysis

#### Studies’ characteristics and quality assessment

A total of 15 articles with 22 studies involving in 1315 cases and 1013 controls were analyzed. The main characteristics of the 22 studies were shown in Table 1. Types of cancer included lung cancer, non-small cell lung cancer (NSCLC), gastric cancer (GC), renal cell cancer (RCC) and pancreatic cancer (PC).The quantitative real-time polymerase chain reaction (qRT-PCR) was used in these studies to test the expression level of miR-486, and the most common reference miRNAs used as the endogenous controls for normalization were RNU6B (U6), miR-39 and miR-16. The quality of the included studies turned out to be generally good and was summarized in Figure 1B.

**Table 1:** The main features of 22 included studies in diagnostic meta-analysis

Study ID	ethnicity	specimen	cas e		con trol		Cancer-type	Control-type	stage	miRNA	Reference miRNA	method	SEN (%)	SPE (%)
			N	age	N	age								
Sromek M 2017	Caucasian	Plasma	61	63	50	57	NSCLC	HC	I-IV	miR-16, miR-205,miR-486	miR-24-3p	qRT-PCR	80.00	95.00
Wang X 2016	Asian	plasma	59	56	59	58	NSCLC	BD	I-III	miR-486,miR-210,CYFRA21-1	miR-16	qRT-PCR	84.70	72.80
Butz H 2016	Caucasian	Urinary Exosome	28	59	18	NA	RCC	HC	NA	miR-126-3p, miR-486-5p	miR-16-5p,miR-106a-5p	qRT-PCR	72.40	60.00
Butz H 2016	Caucasian	Urinary Exosome	81	NA	33	NA	RCC	HC	NA	miR-126-3p, miR-486-5p	miR-16-5p,miR-106a-5p	qRT-PCR	81.30	62.50
Cao Z 2016	Asian	plasma	29	NA	16	NA	PC	BD	I-IV	miR-486-5p,miR-126-3p,miR-106b-3p,miR-938,miR-26b-3p, miR-1285	U6	qRT-PCR	83.90	80.80
Cao Z 2016	Asian	plasma	156	NA	57	NA	PC	BD	I-IV	miR-486-5p,miR-126-3p,miR-106b-3p	U6	qRT-PCR	82.70	84.40
Xu JW 2016	Asian	plasma	156	NA	65	NA	PC	HC	I-IV	miR-486-5p	U6	qRT-PCR	75.00	87.70
Wang LL 2016	Asian	serum	100	59	50	58	GC	HC	I-IV	miR-486	U6	qRT-PCR	76.00	98.00
Yang Y 2016	Asian	plasma	35	NA	30	NA	lung cancer	HC	I-IV	miR-486	cel-miR-39	qRT-PCR	90.00	68.60
Tai M 2016	Asian	blood	110	65	52	66	LAD	HC	I-IV	20miRs1	miR-159a,U6	qRT-PCR	89.10	100.00
Tai M 2016	Asian	blood	143	66	49	66	LAD	HC	I-IV	20miRs1	miR-159a,U6	qRT-PCR	94.40	98.00
Li WS 2016	Asian	plasma	11	59	11	55	NSCLC	HC	I-III	MiR-486	miR-39 ,RNU44	qRT-PCR	90.90	81.80
Jiang X 2015	Asian	plasma	35	NA	30	NA	lung cancer	BD/HC	I-IV	miR-486	cel-miR-39	qRT-PCR	88.50	83.30
Zhu C 2014	Asian	plasma	48	57	102	54	GC	HC	I	miR-16,miR-25,miR-92a,miR-451, miR-486-5p	cel-miR-39	qRT-PCR	72.90	89.20
Zhu C 2014	Asian	plasma	40	54	40	54	GC	HC	I	miR-16,miR-25,miR-92a,miR-451,miR-486-5p	cel-miR-39	qRT-PCR	97.50	87.50
Mozzoni P 2013	Caucasian	plasma	54	69	46	64	NSCLC	BD	I-III	miR-21,miR-486	miR-16	qRT-PCR	87.00	86.50
Shen J 2011	Caucasian, African	plasma	58	68	29	66	NSCLC	HC	I-IV	miR-21,miR-126,miR-210,miR-486-5p	miR-16	qRT-PCR	86.20	96.60
Bianchi F 2011	Caucasian	serum	25	NA	39	NA	LAD	HC	I-IV	34miRs2	6miRs3	qRT-PCR	69.00	84.00
Bianchi F 2011	Caucasian	serum	34	NA	30	NA	NSCLC	HC	I-IV	34miRs2	6miRs3	qRT-PCR	71.00	90.00
Shen Jun 2011	Caucasian, African	plasma	32	66	33	65	NSCLC	BD	I-IV	miR-21,miR-210,miR-486-5p	miR-16	qRT-PCR	75.00	84.90
Shen J 2011	Caucasian, African	plasma	76	68	80	65	NSCLC	BD	I-IV	miR-21,miR-210,miR-486-5p	miR-16	qRT-PCR	76.30	85.00
Yu L 2010	Caucasian, African	sputum	36	68	36	67	LAD	HC	I	miR-486,miR-21,miR-200b,miR-375	U6	qRT-PCR	80.60	91.70
Yu L 2010	Caucasian, African	sputum	64	67	58	65	NSCLC	HC	I-IV	miR-486,miR-21,miR-200b,miR-375	U6	qRT-PCR	70.30	80.00

1:miR-451,miR-1290,miR-636,miR-30c,miR-22-3p,miR-19b,miR-486-5p,miR-20b,miR-93,miR-34b,miR-185,miR-126-5p,miR-93-3p,miR-1274a,miR-142-5p,miR-628-5p,miR-486-3p,miR-425,miR-645,miR-24. 2: let-7a, let-7b, let-7d, miR-103, miR-126, miR-133b, miR-139, miR-140, miR-142, miR-142, miR-148a, miR-148b, miR-17, miR-191, miR-22, miR-223, miR-26a, miR-26b, miR-28, miR-29a, miR-30b, miR-30c, miR-32, miR-328, miR-331, miR-342, miR-374, miR-376a, miR-432, miR-484, miR-486, miR-566, miR-92a and miR-98. 3: miR-197, miR-19b, miR-24, miR-146, miR-15b, miR-19a. Abbreviation: N, number of samples; HC, healthy control; BD, benign disease; SEN, sensitivity; SPE, specificity. NSCLC, non-small cell lung cancer; RCC, renal cell cancer; PC, pancreatic cancer; GC, gastric cancer; LAD, lung adenocarcinoma.

### Pooled diagnostic performance

**Figure 2:**
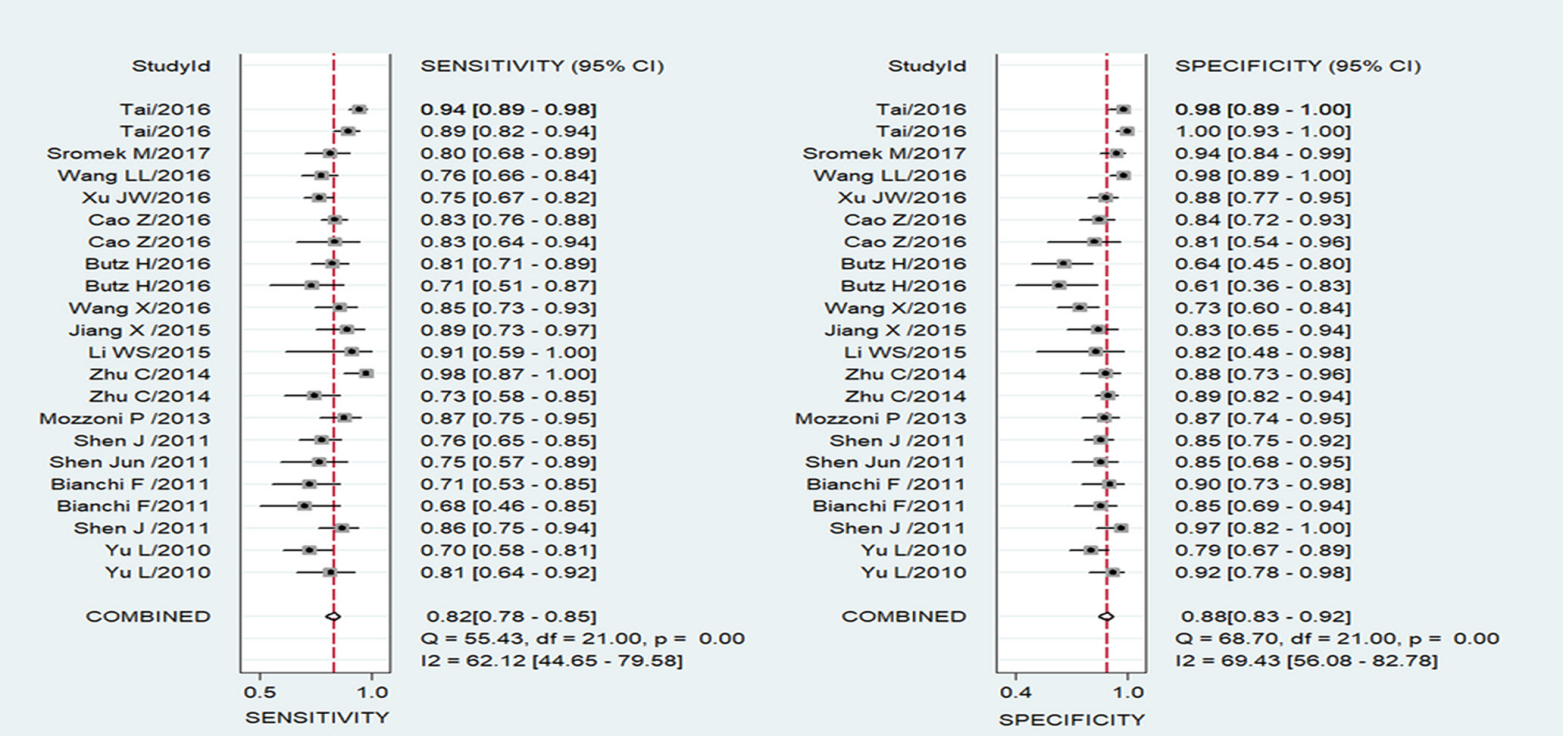
Forest plots of sensitivity and specificity for the cancer diagnosis of miR-486 Both the sensitivity and specificity of each study were showed by square with its 95% Confidence interval showed by the error bars.

**Figure 3:**
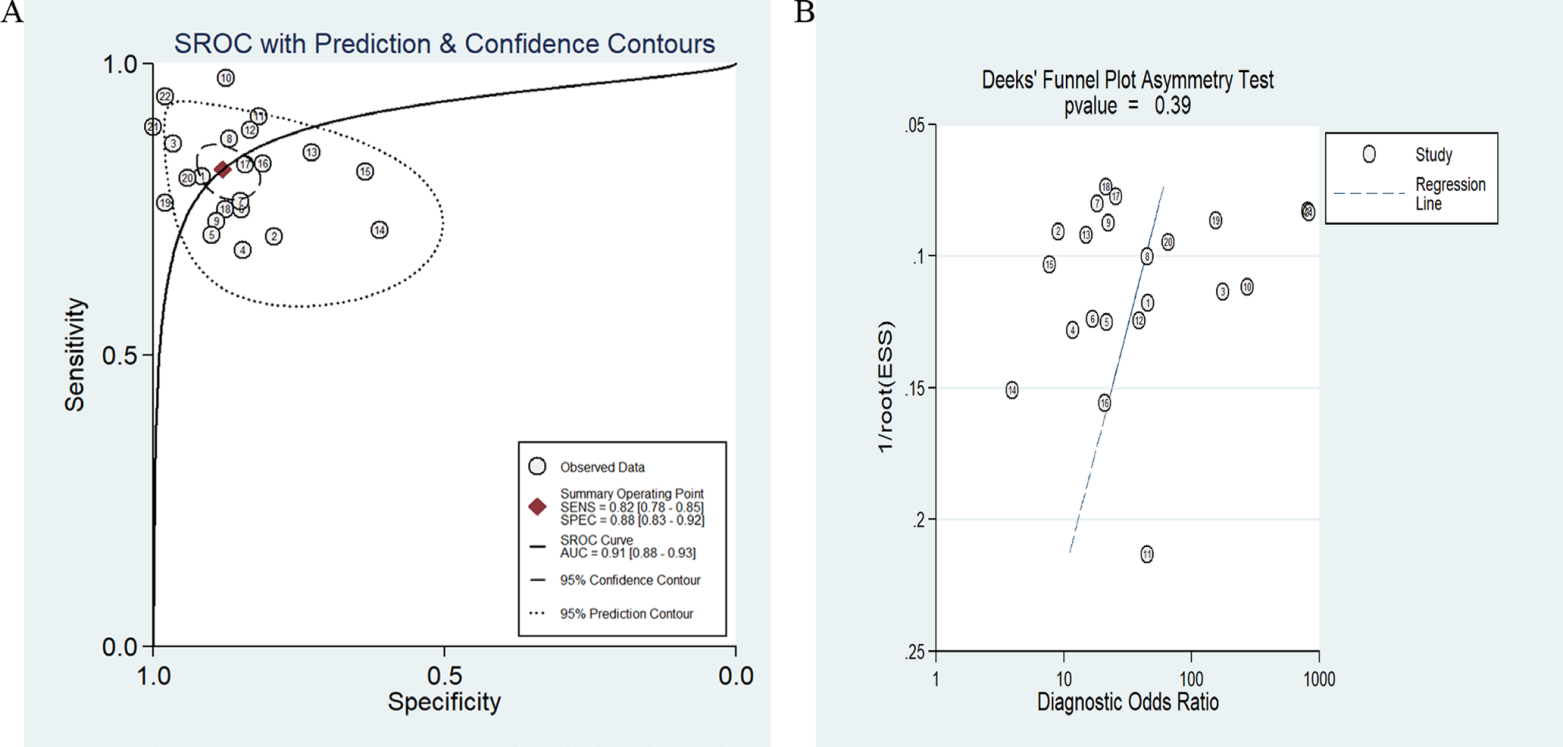
SROC curve of the miR-486 as diagnostic tools for cancer (**A**) and the Deek’s test for assessing the publication bias for miR-486 in the detection of cancer (**B**).

The significant heterogeneity was observed since I^2^ for SEN and SPE were 62.12% (95% CI: 44.65–79.58%) and 69.43% (95% CI: 56.08–82.78%) respectively. Therefore, we used a random-effect model for this analysis. Results showed that overall pooled SEN and SPE for these 22 studies were 82% (95% CI: 78-85%) and 88% (95% CI: 83–92%) respectively to distinguish patients with cancer from the controls (Figure 2). The positive likelihood ratio (PLR) and negative likelihood ratio (NLR) were 6.9 (95% CI: 4.8–9.7) and 0.21 (95% CI: 0.17–0.26) respectively, and the diagnostic odds ratio (DOR) was 33 (95% CI: 20–55). The summary receiver operating characteristic (SROC) curve was performed and the AUC was 0.91 (95% CI: 0.88–0.93) (Figure 3A).

### Publication bias and sensitivity analysis

In this meta-analysis, Deek’s funnel plot asymmetry test was applied to test the probability of publication bias. The funnel plot was symmetry (Figure 3B) and *P* value equaled 0.39, which demonstrated the publication bias didn’t exist in these studies. Sensitivity analysis was also conducted but failed to find the sources of heterogeneity.

### Subgroup analysis and meta-regression analysis

**Figure 4:**
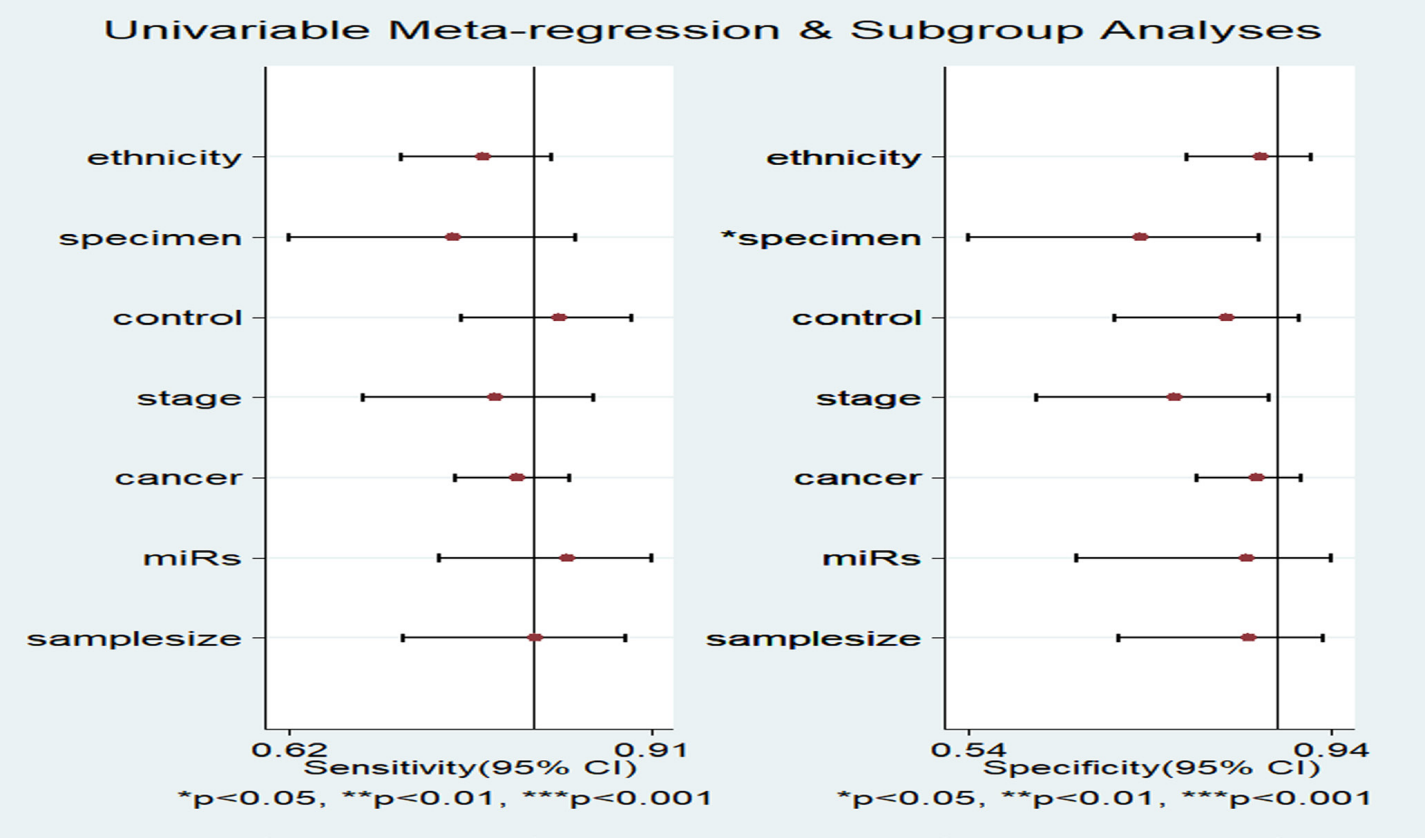
Forest plots for the Meta-regression analysis: sensitivity and specificity The factors included ethnicity, specimen, control, stage, cancer, miRs and sample size.

**Figure 5:**
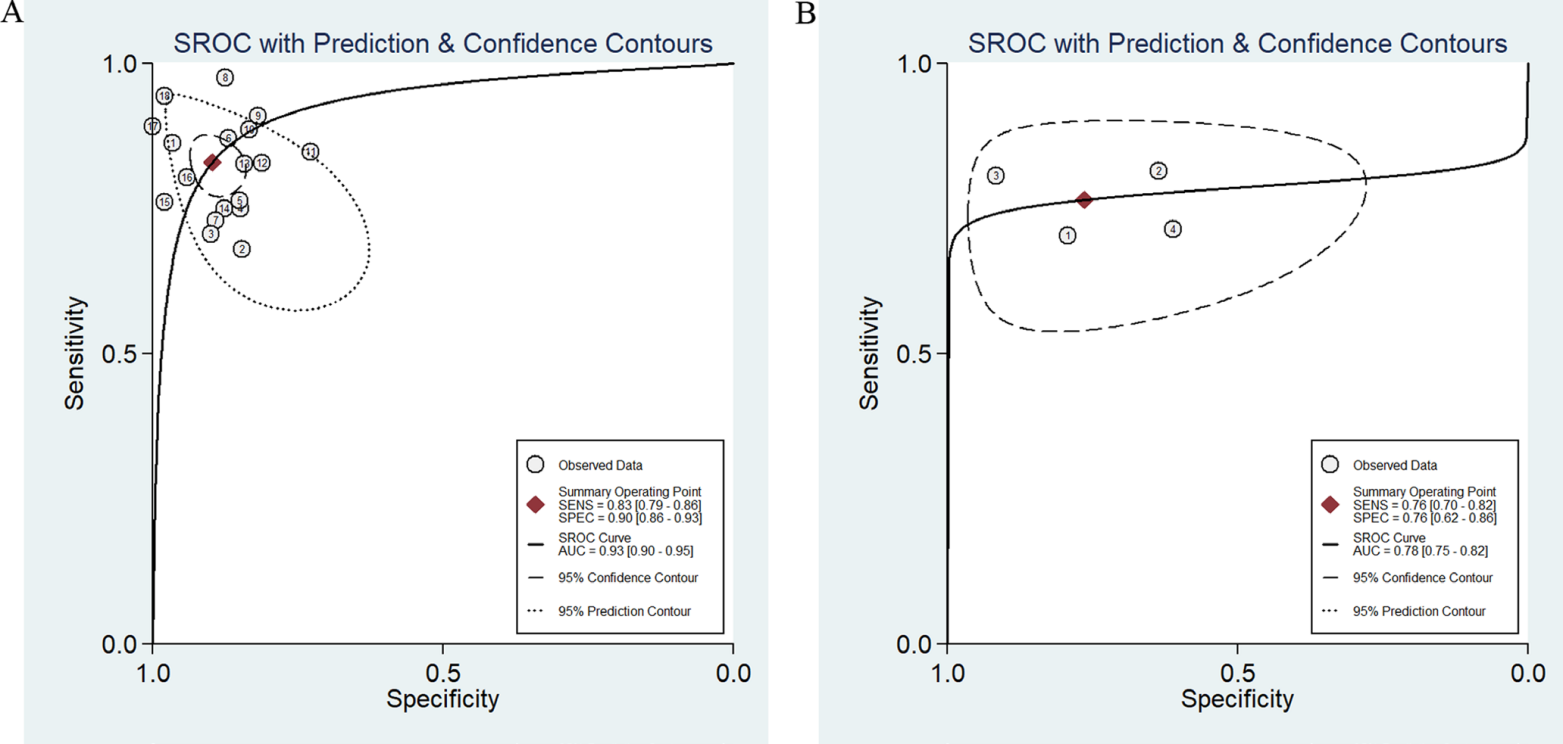
SROC curve of the miR-486 from circulating samples (**A**) and the other specimen (**B**).

**Figure 6:**
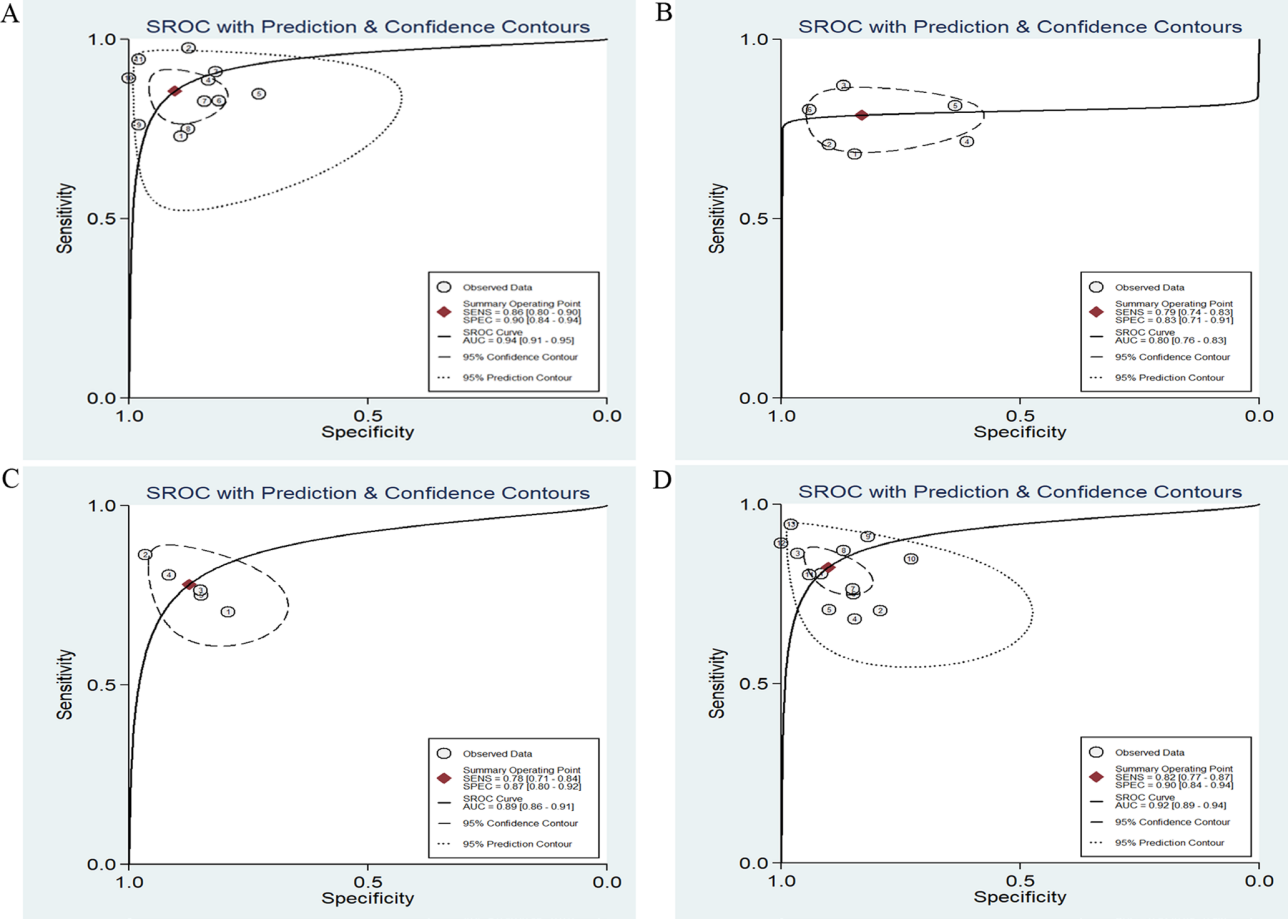
SROC curve of the miR-486 in detection of Asian population (**A**), Caucasian population (**B**) and the Caucasian/ African population (**C**) as well as the SROC curve of miR-486 in the diagnosis of non-small cell lung cancer (**D**).

A multivariate-meta-regression was performed to detect the potential causes of the heterogeneity in both SEN and SPE. The following factors were included: miRs (miR-486 alone or miR-486 with other miRNAs); specimen (circulating or not circulating); ethnicity (Asian, Caucasian or Caucasian/African); control-type (benign disease or healthy controls); stage (early stage or overall stage); cancer-type (lung cancer, gastric cancer, pancreatic cancer and renal cell carcinoma and sample-size (>=100 or < 100). The results demonstrated that specimen (*P* < 0.05) might explain the heterogeneity in SPE shown in Figure 4. Meanwhile, the subgroup analyses were also conducted and the results were presented in Table 2. Subgroup analysis by specimen showed that studies with circulating samples exhibited higher diagnostic accuracy with SEN: 81% (95% CI: 78–84%), SPE: 86% (95% CI: 83-89%) and AUC: 0.90 (95% CI: 0.87-0.92) (Figure 5A) than studies with not circulating samples with the SEN: 76% (95% CI: 70–82%), SPE: 76% (95% CI: 62–86%) and AUC: 0.78 (95% CI: 0.75–0.82) (Figure 5B) respectively. In the subgroup of the ethnicity, the miR-486 obtained a better diagnostic value in the Asian population with the SEN: 86% (95% CI: 80–90%), SPE: 90% (95% CI: 84–94%) and AUC: 0.94 (95% CI: 0.91–0.95) (Figure 6A) when Compared with the Caucasian population with the SEN: 79% (95% CI: 74–83%), SPE: 83% (95% CI: 71–91%) and AUC: 0.80 (95% CI: 0.76–0.83) (Figure 6B) and the Caucasian/African population with SEN: 78% (95% CI: 71–84%), SPE: 87% (95% CI: 80–92%) and AUC: 0.89 (95% CI: 0.86–0.91) (Figure 6C) respectively. With respect to the other types of subgroup analysis, there were no significant differences in the diagnostic accuracy of miR-486 and since there were only 3 studies for early stage and 2 studies that we did not obtain the information about the stage, we only conducted the subgroup analysis for the overall stage of human cancer. However, for the subgroup analysis of NSCLC, it obtained a pretty high diagnostic value with the sensitivity, specificity were 82% (95% CI: 77–87%), 90% (95% CI: 84–94%) respectively as well as the AUC was 0.92 (95% CI: 0.89–0.94) (Figure 6D).

**Table 2:** The subgroup analysis for the selected studies

Subgroups	No.of studies	SEN [95% CI]	SPE [95% CI]	PLR [95% CI]	NLR [95% CI])	DOR [95% CI])	AUC [95% CI]
MiRNA profiling							
single	4	0.78 [0.72–0.83]	0.90 [0.80–0.95]	7.6 [3.9–14.6]	0.24 [0.19–0.32]	31 [15–64]	0.84 [0.81–0.87]
multiple	18	0.83 [0.79–0.86]	0.90 [0.86–0.93]	8.0 [5.7–11.4]	0.19 [0.15–0.24]	42 [25–70]	0.93 [0.90–0.95]
Specimen							
circulating	18	0.81 [0.78–0.84]	0.86 [0.83–0.89]	6.0 [4.7–7.6]	0.22 [0.18–0.26]	28 [20–37]	0.90 [0.87–0.92]
not circulating	4	0.76 [0.70–0.82]	0.76 [0.62–0.86]	3.2 [1.9–5.4]	0.31 [0.23–0.41]	10 [5–21]	0.78 [0.75–0.82]
Ethnicity							
Asian	11	0.86 [0.80–0.90]	0.90 [0.84–0.94]	8.9 [5.1–15.4]	0.16 [0.11–0.23]	55 [25–120]	0.94 [0.91–0.95]
Caucasian	6	0.79 [0.74–0.83]	0.83 [0.71–0.91]	4.7 [2.6–8.3]	0.25 [0.20–0.33]	18 [9–39]	0.80 [0.76–0.83]
Caucasian/African	5	0.78 [0.71–0.84]	0.87 [0.80–0.92]	6.2 [3.7–10.4]	0.25 [0.18–0.35]	24 [11–53]	0.89 [0.86–0.91]
Control–type							
HC	15	0.81 [0.76–0.86]	0.91 [0.84–0.95]	8.7 [5.0–15.1]	0.21 [0.15–0.28]	42 [19–92]	0.91 [0.88–0.93]
BD	6	0.82 [0.78–0.85]	0.83 [0.77–0.87]	4.7 [3.6–6.1]	0.22 [0.18–0.27]	21 [14–32]	0.87 [0.84–0.90]
Cancer type							
NSCLC	13	0.82 [0.77–0.87]	0.90 [0.84–0.94]	8.4 [5.0–14.1]	0.20 [0.15–0.26]	43 [20–92]	0.92 [0.89–0.94]
Sample size							
> = 100	12	0.82 [0.77–0.86]	0.90 [0.82–0.94]	8.1 [4.4–14.7]	0.20 [0.15–0.27]	40 [18–90]	0.90 [0.87–0.92]
< 100	10	0.82 [0.75–0.87]	0.86 [0.81–0.90]	5.8 [4.1–8.2]	0.21 [0.15–0.30]	28 [15–51]	0.91 [0.88–0.93]
Stage							
overall stage	17	0.82 [0.78–0.85]	0.89 [0.85–0.93]	7.8 [5.2–11.6]	0.20 [0.16–0.26]	38 [21–68]	0.91 [0.88–0.93]
Overall studies	22	0.82 [0.78–0.85]	0.88 [0.83–0.92]	6.9 [4.8–9.7]	0.21 [0.17–0.26]	33 [20–55]	0.91 [0.88–0.93]

No: the number of the studies; HC, healthy control; BD, benign pulmonary disease; SEN, sensitivity; SPE, specificity; PLR, positive likelihood ratio; NLR, negative likelihood ratio; DOR, Diagnostic Odds Ratio; AUC, area under the curve.

### Prognostic meta-analysis

#### Studies’ characteristics and quality assessment

A total of 761 participants with cancer from 6 articles on 7 studies were included. The main characteristics of the 7 studies were shown in Table 3 Most studies investigated miR-486 by qRT-PCR. The overall survival (OS), progression-free survival (PFS) and relapse free survival (RFS) were used to evaluate the outcome of the cohorts. Types of the cancer included NSCLC, ESCC, GC and HCC. The results of the studies’ quality assessment were also included in Table 3.

**Table 3:** The main features of 7 included studies in prognostic meta-analysis

Study ID	sex (male/ female	age	ethnicity	specimen	cancer-type	stage	miRNA	reference miRNA	method	outcome	follow-up (month)	HR	ll	ul	NOS
Guo J 2016	25/9	57	Asian	serum	NSCLC	III-IV	miR-486	cel-miR-39	qRT-PCR	PFS	> 8.5	2.04	1.00	4.13	8
Ren CL 2016	137/36	NA	Asian	tissue	ESCC	I-IV	miR-486-5p	Scramble-miR	miRNA-LNA	OS	93.6	4.32	2.62	7.14	7
Ren CL 2016	73/21	NA	Asian	tissue	GC	I-IV	miR-486-5p	Scramble-miR	miRNA-LNA	OS	87.6	2.46	1.35	4.50	7
Li WS 2015	7/4	59	Asian	plasma	NSCLC	I-III	miR-486	cel-miR-39 ,RNU44	qRT-PCR	RFS	24	0.11	0.01	1.06	7
Petriella D 2015	21/9	65	Caucasian	serum	NSCLC	III-IV	miR-486-5p	U6	qRT-PCR	PFS	> 15	5.59	1.32	23.26	7
Wang LM 2015	94/22	54	Asian	serum	HCC	NA	MiR-486-5p	U6	qRT-PCR	RFS	24	1.27	1.12	1.43	7
Hu ZB 2010	222/81	60	Asian	serum	NSCLC	I-III	miR-486	NA	qRT-PCR	OS	61.8	0.50	0.34	0.74	7

Abbreviation: HR, hazard ratio; ll, lower limit; ul, upper Limit; NOS, Newcastle-Ottawa Scale; NSCLC, non-small cell lung cancer; ESCC, esophageal squamous cell carcinoma; GC, gastric cancer; HCC, hepatocellular carcinoma; OS, overall survival; PFS, progression-free survival; RFS, relapse free survival.

### Association between miR-486 and outcomes

**Figure 7:**
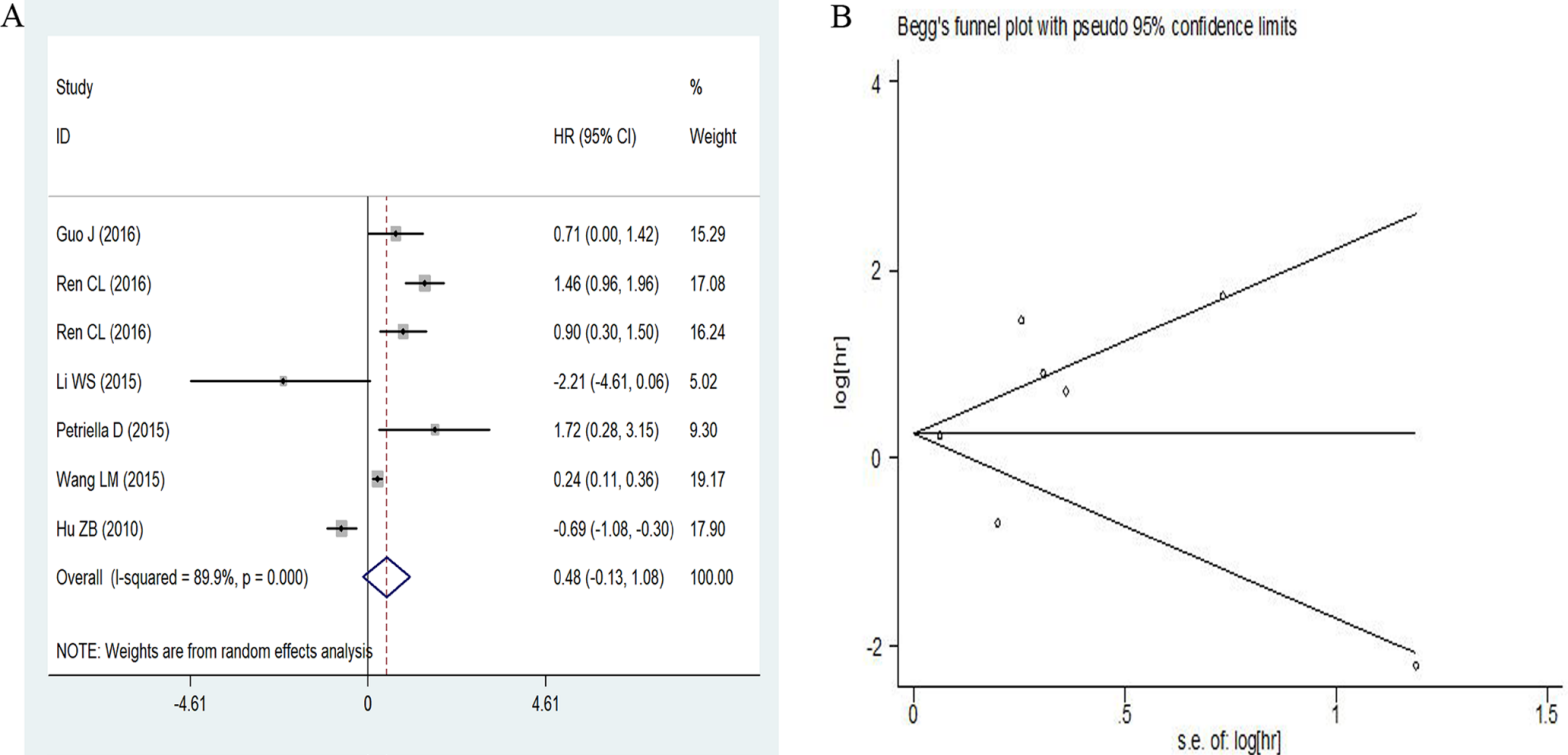
Forest plots of sensitivity and specificity for miR-486 in cancer prognosis (**A**) and the Begg’s funnel plot for the sensitivity analysis (**B**).

A random-effects model was used since the heterogeneity among studies existed (I^2^ = 89.9, *P* = 0.000). The pooled HR (hazard ratio) was 0.48 (95% CI: –0.13–1.08) for low versus high miR-486 expression as shown in Figure 7A. Low miR-486 expression did not increase the risk of poor outcome compared with the high miR-486 expression. And the studies were so few that we did not conduct subgroup analysis for the prognostic meta-analysis.

### Publication bias and sensitivity analysis

Publication bias was checked by Begg’s funnel plot and Egger’s test under the random-effects model (Figure 7B). Although the Begg’s funnel plot seemed asymmetric, the *P* value of the Egger’s regression intercept was 0.676, indicating that there was no obvious publication bias among these studies. The sensitivity analysis was also conducted but failed to find the sources of heterogeneity.

## DISCUSSION

Cancer biomarkers are critical for cancer detection and predicting the outcome as well as choosing the suitable treatment methods. As involving in various biological processes in cancer, miRNA was considered to play a crucial role in cancer diagnosis and prognosis surveillance.

MiR-486 has been reported to be involved in different types of cancer. Research in the mechanism of miR-486 found that the down-regulation of miR-486 could target genes such as ARHGAP5 to inhibit cell migration and invasion *in vitro* and metastasis *in vivo* in lung cancer [6] and it could inhibit cancer cell proliferation, migration and invasive *in vitro* and suppress HCC growth *in vivo* by targeting PIK3R1 [4]. Furthermore, miR-486 might inhibit cell growth of papillary thyroid carcinoma by targeting fibrillin-1 [34] and estrogen receptor-mediated miR-486 could regulate expression of OLFM4 in ovarian cancer [35].

This present meta-analysis aimed to estimate the pooled effect of miR-486 expression on diagnosis and prognosis of human cancer. The diagnostic accuracy of miR-486 was pretty high. The adjusted pooled SEN, SPE and AUC were 82% (95% CI: 78–85%), 88% (95% CI: 83–92%) and 0.91 (95% CI: 0.88–0.93) respectively. With respect to the subgroup analysis, the circulating miR-486 obtained a higher diagnostic value than miR-486 from other specimen. In addition, the accuracy of miR-486 to detect cancer for Asian population was higher than Caucasian or Caucasian/ African population. The pooled AUC of 0.92 (95% CI: 0.89-0.94) indicated that the performance of miR-486 to detect NSCLC was feasible. As for the value of miR-486 on prognosis of cancer, the pooled HR was 0.48 (95% CI: -0.13–1.08) for low versus high miR-486 expression showed that low miR-486 expression did not increase the risk of poor outcome.

To our best knowledge, this meta-analysis was the first one to explore the effect of miR-486 on cancer diagnosis and prognosis. Although we perform this meta-analysis strictly according to the PRISMA guidelines, there were still some limitations that could not be neglected in this meta-analysis. First, the types of cancer included and the studies of each cancer as well as the samples of cases were so few that we could not conduct the subgroup analysis for the prognostic meta-analysis and these limitations might partly contribute to the negative result. Second, the heterogeneity among these studies could not be neglected and some articles might be missed or not be published online that did not be included in this meta-analysis. Third, most studies were from China in the prognostic meta-analysis and the results might just represent the prognostic value of miR-486 on Chinese cancer. Therefore, Studies on the large samples are still demanded to verify our results.

## MATERIALS AND METHODS

### Search strategy

We based our meta-analysis on the Preferred Reporting Items for meta-analyses (PRISMA). We searched PubMed, Cochrane library, Embase, Chinese National Knowledge Infrastructure (CNKI) and Wanfang databases to find all associated articles in order to investigate the potential utility of miR-486 as a diagnostic and prognostic surveillance tool for human cancer. The combination of the Medical Subject Headings (MeSH) and the keywords: (miR-486 or hsa-miR-486 or microRNA-486 or miR486) and (cancer or tumor or carcinoma or neoplasm) was used (updated to September 13, 2017). We also searched reference lists of the reviews aiming at obtaining other acceptable articles.

### Study selection

There was a series of criteria for records inclusion as well as exclusion. For inclusion, records needed to meet the following criteria: 1) Patients of the cases were with cancer; 2) The controls were healthy controls (HC) or with benign diseases (BD); 3) Assess the diagnostic or prognostic value of miR-486 (miR-486-5p); 4) The TP, FP, FN, TN for the diagnosis and HR (hazard ratio) and its 95% CI for the prognosis can be extracted or calculated from the articles. For the exclusion, the criteria as follows: 1) Records that were review, meta-analysis and duplicate publications as well as the records unrelated; 2) Records without complete data or with the same population; 3) Records were about the miR-486-3p.

### Data collection and quality assessment

The data was collected independently by two authors as follows: the first author, year of publication, subject’s demographic characteristics (ethnicity, mean or median age, sample size, testing method of miR-486 and the types of the controls and cancer); types of the specimen; follow-up time and the outcomes; miRNA profiling and the data used for this meta-analysis (SEN, SPE, TP, FP, FN, TN, HR and its 95% CI). All HRs were reformatted as low miR-486 expression versus high miR-486 expression. We assessed the quality of these articles with the Quality Assessment of Diagnostic Accuracy Studies 2 (QUADAS-2) guidelines for the diagnostic records and followed the guidelines of the Newcastle-Ottawa Scale (NOS) for the prognostic publications [36, 37].

### Statistical analysis

We performed the statistical analysis using the STATA 11.0 (STATA-Corp, College Station, TX, version 11.0) software and RevMan 5.3 (version 1.4) software. A bivariate random effect-regression model was applied to assess the pooled SEN [TP/ (FN+TP)], SPE [TN/ (FP+TN)], the positive likelihood ratio (PLR) [(SEN/ (1-SEN)], the negative likelihood ratio (NLR) [(1-SPE)/SPE)], the diagnostic odds ratio (DOR) [PLR/ NLR] and the pooled HR with its 95% Confidence Interval (95% CI) respectively. We also constructed the SROC curve and calculated the AUC value. Simultaneously, we assessed the heterogeneity among the selected studies through the *Q* test and the I^2^ value [38]. The *P* value for the *Q* test less than 0.05 or the I^2^ ≥ 50% demonstrated that there was heterogeneity among the included studies. For the diagnostic meta-analyses, meta-regression analysis and subgroup analysis (grouped by miRNA profiling: single miR-486 and multiple miRNAs including miR-486 and other miRNAs; specimen: circulating and not circulating; ethnicity: Asian, Caucasian and Caucasian/African; control-type: benign disease and healthy controls; stage: early stage and not only early stage; cancer-type: lung cancer, gastric cancer, pancreatic cancer and renal cell carcinoma and sample size: >= 100 and < -100) were used to identify the potential sources of the heterogeneity and the Deek ’s funnel plot asymmetry test was also applied to explore the publication bias, with the *P* value less than 0.01 considered significant [39]. As for the prognostic meta-analyses, Begg’s and Egger’s tests were selected to evaluate the included studies for the possibility of publication bias. Finally, the sensitivity analysis was conducted to explore the possible sources of heterogeneity for both the diagnosis and the prognosis meta-analysis.

## CONCLUSIONS

This was the first meta-analysis to confirm the potential value of miR-486 on cancer diagnosis and prognosis. The expression of miR-486 might be an effective biomarker for detection of human cancer.

## Abbreviations

miR-486: has-miR-486/ has-miR-486-5p
miRNA: microRNA
mRNA: messenger RNA
3’-UTR: 3’-untranslated region
NSCLC: non-small cell lung cancer
HCC: hepatocellular carcinoma
ESCC: esophageal squamous cell carcinoma
PC: pancreatic cancer
GC: gastric cancer
RCC: renal cell cancer
PRISMA: preferred Reporting Items for meta-analysis
HC: Healthy Control
BD: benign disease
SEN: sensitivity
SPE: specificity
SROC: summary receiver operating characteristic
AUC: the area under the SROC curve
CNKI: Chinese national knowledge infrastructure
PLR: positive likelihood ratio
NLR: negative likelihood ratio
DOR: diagnostic odds ratio
TP: true positive
FP: false positive
FN: false negative
TN: true negative
qRT-PCR: quantitative real-time polymerase chain reaction
HR: hazard ratio
OS: overall survival
PFS: progression-free survival
RFS: relapse free survival
QUADAS-2: the Quality Assessment of Diagnostic Accuracy Studies-2
NOS: the Newcastle-Ottawa Scale
